# A Contemporary Mini-Review of Interprofessional Education and Technology-Assisted Management of Dental Emergencies in the Emergency Department

**DOI:** 10.3390/healthcare14040544

**Published:** 2026-02-22

**Authors:** Zanab Malik, Tony Skapetis

**Affiliations:** 1 School of Health Sciences (Oral Health), College of Health, Medicine and Wellbeing, The University of Newcastle, Callaghan, NSW 2308, Australia; 2 NSW Health, South Western Sydney Local Health District, Campbelltown, NSW 2560, Australia; 3 School of Dentistry, Faculty of Medicine and Health, The University of Sydney, Surry Hills, NSW 2010, Australia; tony.skapetis@sydney.edu.au; 4 NSW Health, Western Sydney Local Health District, Westmead, NSW 2145, Australia; 5 School of Health Sciences, Western Sydney University, Penrith, NSW 2751, Australia

**Keywords:** emergency service, hospital, emergency treatment, dental care, education, professional, technology

## Abstract

Background: Dental emergencies are increasing in frequency. Numerous studies have reported minimal knowledge and/or skills by emergency department staff regarding dental emergencies. The COVID-19 pandemic has prompted a paradigm shift in emergency dental care management away from traditional management approaches. However, there have been no reviews of contemporary literature pertaining to either technology-assisted or interprofessional education and dental emergency management in the emergency department setting. This mini-review aimed to synthesise current evidence of interprofessional education, utilising technology-assisted modalities, for the management of dental emergencies in hospital emergency departments. Methods: A comprehensive search was carried out across four electronic databases, Medline, Embase, CINAHL, and Google Scholar from 2018 to 2025. Results: A total of three papers were identified and included in the mini-review. Two of the three papers addressed the subject of dental emergencies in the emergency department as a primary finding. Discussion: Included papers were of low-quality evidence and referenced simulation-based education, tele-dentistry, and artificial intelligence as contemporary approaches relating to dental emergency management. Conclusions: This mini-review revealed minimal advances in contemporary approaches relating to both the use of technology-assisted modalities and interprofessional education for the management of dental emergencies within the hospital emergency department setting. This review provides a timely literature update for both the medical and dental professions and identifies a large gap in research surrounding this topic.

## 1. Introduction

Dental emergencies are increasing in frequency, especially in low socio-economic and minority groups and those living with special needs [1,2,3]. Dental-related emergencies, including tooth pain, infection, traumatic dental injuries, and post-operative dental complications, frequently present to general hospital emergency departments (ED) instead of dental practices [4,5]. There may be multifactorial causes for this, for example, availability of dental services after hours, severity of dental emergency, dental anxiety resulting in deferral of preventive treatment, transport issues, or cost [4]. However, significant barriers such as long waiting times, dental anxiety, and medical complexity can complicate presentations in the ED. Whilst the aim for management of dental emergencies is stabilisation and subsequent referral to the dental team for definitive management, appropriate initial diagnosis and treatment reduces negative effects such as patient suffering and potential further dental expenses for individuals and health systems [6]. It is therefore prudent that both dental and medical professions are unified on what expectations are for the dental management of emergencies in the ED.

There has been documented low or insufficient knowledge globally regarding the emergency management of traumatic dental injuries by non-dental health care professionals [7]. Among ED physicians, data suggests a lack of confidence in the management of dentofacial emergencies [8]. Numerous studies have reported minimal knowledge and/or skills in this regard, largely due to the lack of adequate dental education being embedded within current medical curricula or training, and exposure to dentofacial emergencies [8].

The COVID-19 pandemic prompted a paradigm shift in emergency dental care management, and traditional management approaches have required reassessment within dental settings. There is advocacy for innovative solutions and adaptive strategies in emergency dental care, with a commitment to interprofessional collaboration, evidence-based practice, and patient safety [9]. The World Health Organisation reports interprofessional education occurs when “students from two or more professions learn about, from, and with each other to enable effective collaboration and improve health outcomes” [10]. Interprofessional education has also been shown to improve patient satisfaction, and studies have shown how this can be incorporated into emergency medicine training with the aim of promoting the working relationships between the medical and dental profession [11,12].

Several studies have investigated technological advances in the field of medical emergency training in dental clinic settings. This has included the use of artificial intelligence, role-play simulations, or virtual reality and augmentative reality modalities [13]. For the purposes of this paper, we have included these under the umbrella term “technology-assisted” management approaches.

A majority of studies in one systematic review found significant improvements in dental educational outcomes and positive appraisal of virtual technologies [14]. Despite the recognised importance of early and effective management of dental emergencies, there is a paucity of literature examining contemporary technology-assisted and interprofessional education for hospital ED clinicians. This mini-review was therefore conducted to synthesise current evidence, identify gaps in training and educational strategies, and inform future interventions to enhance clinician preparedness and patient care. The aim of this mini-review was to evaluate contemporary evidence for interprofessional education and technology-assisted modalities in the management of dental emergencies within hospital-based EDs.

## 2. Materials and Methods

Ethics approval was not required as this is a mini-review of previously published literature. A comprehensive search was carried out across four relevant electronic databases: Medline, Embase, CINAHL, and Google Scholar. These databases were selected after discussion with a medical librarian for medical and dental literature with an educational focus. Papers dating from 2018 to 2025, published in English, those with study design, study participants, and intervention type (interprofessional education or technology-assisted training relevant to dental emergencies) were included, with the aim to answer the following research question: What is the contemporary evidence for interprofessional education and use of technology-assisted modalities in dental emergency management within hospital-based EDs?

In addition to peer-reviewed journal articles, grey literature, including conference abstracts were considered to ensure comprehensive coverage of the available evidence. Papers were excluded if the focus was on dental rather than general hospital EDs, non-relevant educational interventions, or non-English publications. Controlled vocabulary terms, including MeSH terms in Medline and equivalent subject headings in other databases, were used where available in combination with free-text keywords to account for variations in indexing across medical, dental, and educational literature. Boolean operators (AND, OR) and truncations were applied to combine search terms and optimise retrieval of relevant studies, with search strategies adapted to the indexing requirements of each database. The search strategy included the terms ‘interprofessional education’, ‘technology advancement’, ‘technology-assisted’, ‘artificial intelligence’, ‘AI’, ‘simulation training’, ‘simulation education’, ‘simulation learning’, ‘virtual reality’, ‘VR’, ‘augmented reality’, ‘dental emergencies’, ‘emergency medicine’, ‘medical hospital’, ‘acute hospital’ and ‘general hospital’ to elicit results regarding dental emergency education and technological advancement in the ED (see Table S1). The research team performed title and abstract screening of the articles identified from the electronic search. PRISMA (Preferred Reporting Items for Systematic Reviews and Meta-Analyses) principles were followed to guide transparent reporting of study identification and selection prior to proceeding to a mini-review of full-text papers for inclusion/exclusion criteria. Titles and abstracts identified through the electronic search were independently screened by two reviewers, followed by full-text assessment against predefined inclusion and exclusion criteria. Any discrepancies were resolved through discussion and consensus, and no further consultation from an additional reviewer was required.

The review methodology consisted of searching for contemporary approaches or technological advancements, interprofessional education, and future directions with regard to dental emergency management in the ED. Given the sparsity of available literature, this was undertaken with the intent to map all published contemporary research and to establish whether a future systematic review may be warranted.

## 3. Results

**Figure 1:**
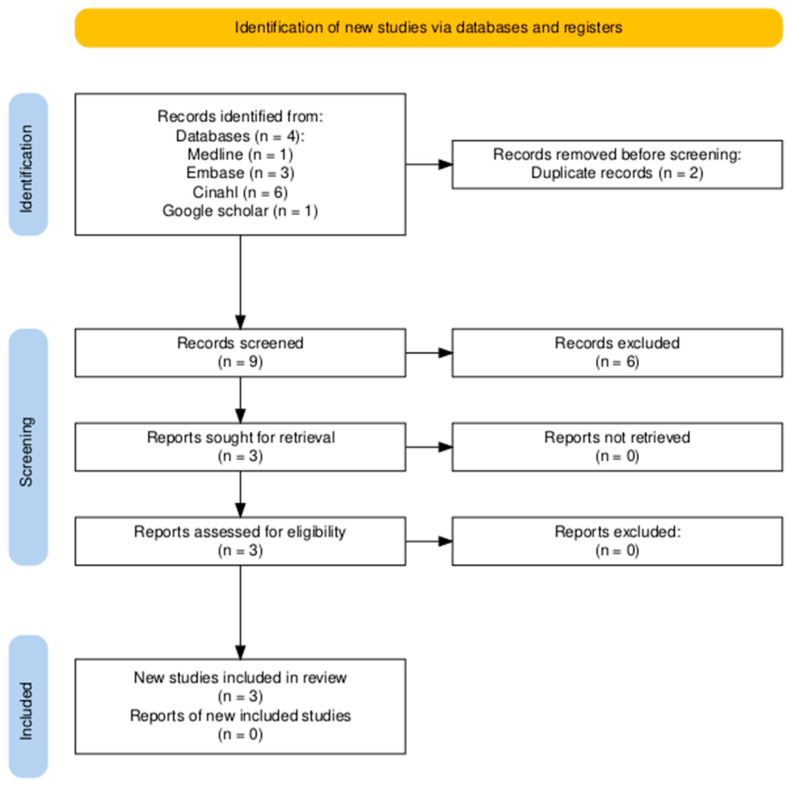
PRISMA flow diagram, which depicts the number of records identified, screened, excluded, and included in the final synthesis.

The searches across the four databases initially identified nine records. Following title and abstract screening, six studies were excluded for not meeting inclusion criteria (publication date, topic relevance), leaving three studies for inclusion in this mini-review, comprising two full-text articles and one conference abstract [15,16,17] (see Figure 1). A general literature search regarding interprofessional education within the ED was also undertaken; however, no further studies were included as no dental education was referenced. The included studies and their characteristics were summarised in Table 1.

The papers included one feasibility study, one cross-sectional study, and one editorial. The limited number of papers contained small or no sample sizes and were of very low to low quality evidence due to their risk of bias and limited generalisability. The contemporary approaches referenced were tele-dentistry and simulation-based education from the included papers, with an additional reference to artificial intelligence as an advanced technology. The full text of one paper was a conference abstract journal issue, which was analysed, and hence the country of origin could not be established, or if there had been dental input to the simulation-based education involving ED physicians [16].

Two of the three papers [15,16] addressed the subject of dental emergencies in the ED as a primary finding, while the editorial addressed this as a secondary finding. The reference to artificial intelligence was specific to only limited examples of dental emergencies used in the text, limiting the generalisability to use in the ED, where traumatic and non-traumatic dental injuries may present.

## 4. Discussion

There is a paucity of literature examining contemporary technology-assisted and interprofessional education in the management of dental emergencies within the ED. This limited evidence highlights that, despite the recognised importance of early and effective management of dental trauma and emergencies, there has been minimal advancement in integrating dental education into medical curricula and ED training programmes in recent years. Consequently, a synthesis of existing studies was warranted to identify current gaps, inform future educational initiatives, and guide research into effective strategies for improving clinician preparedness and patient care in ED settings.

The literature has discussed the need for enhanced education, with previous proposals for the introduction of dental trauma into medical curricula at the tertiary level to ensure early management and improved patient outcomes [18]. Adequate knowledge allows for appropriate examination and triaging of dental emergencies within the ED. Improved inter-professional collaboration at the tertiary level between medical and dental schools, or continuing professional development courses for ED staff, has been recommended [19]. Systematic reviews reveal that the most effective continuing medical education is that with multiple media and multiple educational exposures for reinforcement [20].

In Australia, educational initiatives such as multi-modal workshops have been implemented and evaluated with good success, and statewide websites such as the Agency of Clinical Innovation; https://aci.health.nsw.gov.au (accessed on 1 January 2026) propose clinical guidelines accessible to ED staff. However, qualitative data recommend caution with online resources, as professional ED staff value personal engagement, particularly given the sensitive dynamics of emergency medicine [21].

The premise underpinning interprofessional education is to work collaboratively to improve patient care [22]. Recommendations include validated guidelines and training resources for ED clinicians, guided by dental professional input, to prevent patients with dental emergencies from receiving suboptimal care, especially where onsite specialist oral and maxillofacial support is unavailable [8]. There is also a reliance on dental professional input and appropriate resourcing of dental materials and instruments to enable emergency dental procedures [19].

This mini-review reinforces the need for interprofessional collaboration and technology-assisted solutions in ED settings. Dental professionals and ED clinicians may benefit from integrating digital triage tools, teleconsultations, and decision-support frameworks to support timely and appropriate management of dental emergencies. Regular interprofessional education, combining multiple modalities and reinforcement strategies, can enhance clinician confidence and competence, reduce mismanagement, and improve patient outcomes. Clinically, these strategies may reduce unnecessary hospital visits, optimise patient experience, and ensure that dental emergencies are managed efficiently, even in resource-limited settings.

### 4.1. Study Strengths and Limitations

This mini-review has several strengths. It provides a timely synthesis of contemporary evidence on interprofessional education and technology-assisted modalities for the management of dental emergencies in hospital-based EDs, a topic for which published research is scarce. The inclusion of multiple electronic databases and grey literature, as well as the use of a systematic screening process guided by PRISMA, enhances the comprehensiveness and transparency of the review. Additionally, key study characteristics and outcomes are made available in a Supplementary File to facilitate reproducibility.

However, this review also has limitations. Only English-language publications were included, which may have introduced selection bias and led to the exclusion of relevant studies in other languages. Furthermore, the literature on interprofessional education and technology-assisted management of dental emergencies in hospital-based EDs is extremely limited. Specific limitations of this review include the small number of included studies, the low level of evidence, the reliance on narrative synthesis, and the restricted generalisability of the findings. Consequently, findings should be interpreted with caution, and further research is needed to strengthen the evidence base in this area.

### 4.2. Future Directions

Advances in dental medicine and technology can play an innovative role in improving the management of dental emergencies in EDs globally. Notably, there is a growing body of literature on technology-assisted management of medical emergencies in dental practice settings and educational initiatives in tertiary education. Web-based applications, tele-dentistry, and simulation-based education have been evaluated for feasibility [6]. Tele-dentistry platforms with real-time video consultations, virtual dental assessments with intra-oral cameras, and remote monitoring could be adapted to ED settings [9]. From these initiatives, similar initiatives can be trialled for ED settings. Artificial intelligence and machine learning algorithms may assist triaging and decision support for ED clinicians, warranting further development and research. Overall, enhanced workflows, interprofessional collaboration, and innovative teaching methods are needed to bridge current gaps in education and ensure consistent, high-quality care for patients presenting with dental emergencies [23].

## 5. Conclusions

This mini-review highlights the very limited contemporary evidence on interprofessional education and technology-assisted management of dental emergencies in hospital-based emergency departments. The few available studies, comprising small sample sizes and low-quality evidence, suggest that educational interventions and digital tools may have the potential to improve clinician preparedness and patient care, but definitive conclusions cannot be drawn. These findings underscore the need for further research to evaluate effective educational strategies, develop validated training resources, and explore technology-assisted solutions to enhance interprofessional collaboration in emergency settings.

## Figures and Tables

**Table 1:** Characteristics of included studies (*n* = 3) [15,16,17].

Author (Year of Publication)	Sample Size	Study Location	Methodology	Study Population	Primary/Secondary Findings of Research Paper with regard to Dental Emergency Management in the Emergency Department	Key Findings and Recommendations Related to Interprofessional Education or Technological Advances in the Emergency Department
Abdelrahim et al., 2020	13	USA	Feasibility study	Participants with non-traumatic dental conditions	Primary	Use of tele-dental intervention in the Emergency department/urgent care settings demonstrated potential feasibility in addressing the non-traumatic dental conditions presenting in the ED.
Sharma et al., 2023	27	USA	Cross-sectional study	Postgraduate ED residents	Primary	Simulation-based workshops, including performance of facial nerve blocks; post-extraction bleeding management; tooth preservation and reimplantation; tooth splinting; and treatment of dental fractures, resulted in increased confidence, knowledge, and skills of physician residents.
Veseli et al., 2023	N/A	N/A	Editorial	N/A	Secondary	The use of ChatGPT (GPT-3.5) for the management of dental treatment (non-traumatic) cases for patients, in particular, who have emigrated abroad, may present as an auxiliary tool; however, there was no mention of use in the emergency department.

## Data Availability

The original contributions presented in this study are included in the article/Supplementary Material. Further inquiries can be directed to the corresponding author.

## Supplementary Materials

The following supporting information can be downloaded at: https://www.mdpi.com/article/10.3390/healthcare14040544/s1, Table S1: Search strategy.
