# Supplementary material for: A Contemporary Mini-Review of Interprofessional Education and Technology-Assisted Management of Dental Emergencies in the Emergency Department
Source: Healthcare (Basel). 2026 Feb 22;14(4):544. doi: 10.3390/healthcare14040544 (PMC12941235; doi:10.3390/healthcare14040544)
Supplement: Supplementary file 1 [file healthcare-14-00544-s001.zip › healthcare-4104487-supplementary.pdf]

Table S1: Search strategy:

# ▲	Searches	Results
1	interprofessional education.mp.	5693
2	technolog*.mp.	1098363
3	advance*.mp.	1666216
4	artificial intelligence.mp.	108112
5	simulation.mp.	612520
6	virtual reality.mp.	39042
7	augmented reality.mp.	7254
8	2 OR 3 OR 4 OR 5 OR 6 OR 7	138755
9	dental emergenc*.mp.	650
10	emergency medicine.mp.	67628
11	emergency department.mp.	203262
12	general hospital.mp.	85254
13	1 AND 8	1429
14	9 AND 12	4
15	1 AND 14	0
16	8 AND 14	1
17	1 AND 10	65
18	8 AND 9	28
19	9 AND 11	75

20	1 AND 19	0
21	8 AND 19	3
